# Compensatory-reserve-weighted intracranial pressure versus intracranial pressure for outcome association in adult traumatic brain injury: a CENTER-TBI validation study

**DOI:** 10.1007/s00701-019-03915-3

**Published:** 2019-05-03

**Authors:** Frederick A. Zeiler, Ari Ercole, Manuel Cabeleira, Erta Beqiri, Tommaso Zoerle, Marco Carbonara, Nino Stocchetti, David K. Menon, Peter Smielewski, Marek Czosnyka, CENTER-TBI High Resolution ICU Sub-Study Participants and Investigators, Audny Anke, Ronny Beer, Bo-Michael Bellander, Andras Buki, Giorgio Chevallard, Arturo Chieregato, Giuseppe Citerio, Endre Czeiter, Bart Depreitere, George Eapen, Shirin Frisvold, Raimund Helbok, Stefan Jankowski, Daniel Kondziella, Lars-Owe Koskinen, Geert Meyfroidt, Kirsten Moeller, David Nelson, Anna Piippo-Karjalainen, Andreea Radoi, Arminas Ragauskas, Rahul Raj, Jonathan Rhodes, Saulius Rocka, Rolf Rossaint, Juan Sahuquillo, Oliver Sakowitz, Ana Stevanovic, Nina Sundström, Riikka Takala, Tomas Tamosuitis, Olli Tenovuo, Peter Vajkoczy, Alessia Vargiolu, Rimantas Vilcinis, Stefan Wolf, Alexander Younsi

**Affiliations:** 1 Division of Anaesthesia, Addenbrooke’s Hospital, University of Cambridge, Cambridge, UK; 2 Department of Surgery, Rady Faculty of Health Sciences, University of Manitoba, Winnipeg, MB R3A 1R9 Canada; 3 Brain Physics Laboratory, Division of Neurosurgery, Addenbrooke’s Hospital, University of Cambridge, Cambridge, UK; 4 Department of Physiopathology and Transplantation, Milan University, Milan, Italy; 5 Neuro ICU Fondazione IRCCS Cà Granda Ospedale Maggiore Policlinico Milan, Milan, Italy; 6 Institute of Electronic Systems, Warsaw University of Technology, Warsaw, Poland

**Keywords:** Compensatory reserve, Intracranial pressure, Outcome, Weighted ICP

## Abstract

**Background:**

Compensatory-reserve-weighted intracranial pressure (wICP) has recently been suggested as a supplementary measure of intracranial pressure (ICP) in adult traumatic brain injury (TBI), with a single-center study suggesting an association with mortality at 6 months. No multi-center studies exist to validate this relationship. The goal was to compare wICP to ICP for association with outcome in a multi-center TBI cohort.

**Methods:**

Using the Collaborative European Neuro Trauma Effectiveness Research in TBI (CENTER-TBI) high-resolution intensive care unit (ICU) cohort, we derived ICP and wICP (calculated as wICP = (1 − RAP) × ICP; where RAP is the compensatory reserve index derived from the moving correlation between pulse amplitude of ICP and ICP). Various univariate logistic regression models were created comparing ICP and wICP to dichotomized outcome at 6 to 12 months, based on Glasgow Outcome Score—Extended (GOSE) (alive/dead—GOSE ≥ 2/GOSE = 1; favorable/unfavorable—GOSE 5 to 8/GOSE 1 to 4, respectively). Models were compared using area under the receiver operating curves (AUC) and *p* values.

**Results:**

wICP displayed higher AUC compared to ICP on univariate regression for alive/dead outcome compared to mean ICP (AUC 0.712, 95% CI 0.615–0.810, *p* = 0.0002, and AUC 0.642, 95% CI 0.538–746, *p* < 0.0001, respectively; no significant difference on Delong’s test), and for favorable/unfavorable outcome (AUC 0.627, 95% CI 0.548–0.705, *p* = 0.015, and AUC 0.495, 95% CI 0.413–0.577, *p* = 0.059; significantly different using Delong’s test *p* = 0.002), with lower wICP values associated with improved outcomes (*p* < 0.05 for both). These relationships on univariate analysis held true even when comparing the wICP models with those containing both ICP and RAP integrated area under the curve over time (*p* < 0.05 for all via Delong’s test).

**Conclusions:**

Compensatory-reserve-weighted ICP displays superior outcome association for both alive/dead and favorable/unfavorable dichotomized outcomes in adult TBI, through univariate analysis. Lower wICP is associated with better global outcomes. The results of this study provide multi-center validation of those seen in a previous single-center study.

**Electronic supplementary material:**

The online version of this article (10.1007/s00701-019-03915-3) contains supplementary material, which is available to authorized users.

## Introduction

Intracranial pressure (ICP) is well known to be associated with outcome in adult traumatic brain injury (TBI), with higher sustained levels of ICP linked to worse global outcome, and particularly higher mortality [1, 3, 5, 9, 11]. Various studies to date have documented such associations, with defined treatment thresholds providing one of the main therapeutic targets in the critical care management of the moderate/severe TBI patient [3, 9].

Recent single-center retrospective work has suggested a newer variant of ICP in adult TBI, termed compensatory-reserve-weighted ICP [2, 6]. Using information from the continuously updating cerebral compensatory reserve index (RAP) [2, 4, 12], derived from the moving correlation coefficient between pulse amplitude of ICP (AMP) and ICP, one can derive a “weighted” ICP (wICP) as wICP = (1 − RAP) × ICP [2, 6]. Such characteristics of wICP have been described in detail within this previous work [2], with a strong association between wICP and mortality being seen on descriptive analysis.

However, despite the promise of wICP as a combined measure of ICP and compensatory reserve in adult TBI, these conclusions have been based on data from a single-center series [2, 6]. This limitation stems from the complexity of the data required for analysis. With the completion of the Collaborative European Neuro Trauma Effectiveness Research in TBI (CENTER-TBI) study [8], the high-resolution intensive care unit (ICU) cohort has provided a multi-center data set with high-frequency digital physiologic signals. The goal of this study is to compare the association between ICP and wICP with global patient outcome in adult TBI, using the CENTER-TBI high-resolution ICU cohort.

## Methods

### Patient population

All patients from the multi-center CENTER-TBI high-resolution ICU cohort were included for this study. These patients were prospectively recruited during the periods of January 2015 to December 2017. A total of 21 centers in the European Union (EU) recruited patients for this cohort. All patients were admitted to ICU for their TBI during the course of the study, with high-frequency digital signals recorded from their ICU monitors during the course of their ICU stay. All patients suffered predominantly from moderate to severe TBI (moderate = Glasgow Coma Score (GCS) 9 to 12, and severe = GCS of 8 or less). A minority of patients suffered from non-severe TBI, with subsequent early deterioration leading to ICU admission for care and monitoring. All patients in this cohort had invasive ICP monitoring conducted in accordance with the BTF guidelines [3].

### Data collection

As part of recruitment to the multi-center high-resolution ICU cohort of CENTER-TBI [8], all patients had demographics prospectively recorded. Similarly, all patients had high-frequency digital signals from ICU monitoring recorded throughout their ICU stay, with the goal of initiating recording within 24 h of injury. All digital ICU signals were further processed (see the “Signal acquisition”/“Signal processing” section). For the purpose of this study, the following admission demographic variables were collected: age, sex, admission Glasgow Coma Scale (GCS; total and motor), and admission pupillary response (bilaterally reactive, unilateral reactive, bilateral unreactive). Only non-imputed raw data was used for the purpose of this study, given the CENTER-TBI study-wide imputation for missing data is still ongoing and will be the focus of various other publications and studies. Data was accessed on Sept 16, 2018, via Opal database software [7].

### Signal acquisition

Arterial blood pressure (ABP) was obtained through either radial or femoral arterial lines connected to pressure transducers (Baxter Healthcare Corp. CardioVascular Group, Irvine, CA). ICP was acquired via an intra-parenchymal strain gauge probe (Codman ICP MicroSensor; Codman & Shurtleff Inc., Raynham, MA), parenchymal fiber optic pressure sensor (Camino ICP Monitor, Integra Life Sciences, Plainsboro, NJ, USA; https://www.integralife.com/) or external ventricular drain. All signals were recorded using digital data transfer or digitized via an A/D converter (DT9801; Data Translation, Marlboro, MA), where appropriate, sampled at frequency of 100 Hz or higher, using the ICM+ software (Cambridge Enterprise Ltd., Cambridge, UK, http://icmplus.neurosurg.cam.ac.uk) or Moberg CNS Monitor (Moberg Research Inc., Ambler, PA, USA) or a combination of both. Signal artifacts were removed using both manual and automated methods prior to further processing or analysis.

### Signal processing

Post-acquisition processing of the above signals was conducted using ICM+. CPP was determined as CPP = MAP − ICP. AMP was determined by calculating the fundamental Fourier amplitude of the ICP pulse waveforms over a 10-s window, updated every 10 s. Ten-second moving averages (updated every 10 s to avoid data overlap) were calculated for all recorded signals: ICP, ABP (which produced MAP), AMP, and CPP.

RAP, the continuous index of cerebral compensatory reserve was derived as the moving correlation coefficient between 30 consecutive 10-s mean windows of the parent signals (AMP and ICP), updated every minute. Finally, compensatory-reserve-weighted ICP (wICP) was created for each minute-by-minute observation via the following previously described method: wICP = (1 − RAP) × ICP.

Data were provided in minute-by-minute comma separated variable sheets for the entire duration of recording for each patient.

The basis for interpretation of wICP has been previously published [2], and a brief summary is provided here: RAP shows values around 0 when ICP is low and compensatory reserve is good (linear part of pressure-volume curve). RAP increases to +1 at higher ICP indicating poor compensatory reserve (exponential part of pressure-volume curve). Such a state is usually seen almost all the time after severe TBI. When ICP increases to very high values, it provokes gradual collapse of cerebral arterial bed and rapid decrease of cerebral blood flow. RAP decreases towards zero or negative (deflection of pressure-volume curve). This “very high value of ICP” leading to vascular collapse is individual and cannot be substituted by any cohort-based average, like 20 or 25 mmHg. Multiplying ICP by (1 − RAP) would magnify these values of ICP when it interferes with integrity of cerebral blood flow, by taking into account the cerebral compensatory reserve during the calculation.

### Data processing

Grand (i.e., the entire recording period) mean values of all physiologic variables were calculated per patient. In addition, post-ICM+ processing of RAP physiologic data occurred in R (R Core Team (2016). R: A language and environment for statistical computing. R Foundation for Statistical Computing, Vienna, Austria. URL https://www.R-project.org/), in keeping with our recent single-center retrospective study on RAP in adult TBI, using the *flux* package. Area under the curve for RAP (RAP AUC) was determined for each patient by integrating the RAP signal over time via a sequential linear interpolation method within R using minute-by-minute data. RAP AUC over time was calculated for RAP thresholds of 0 and + 0.4. This is in keeping with previous work on the association between RAP and admission brain imaging characteristics [12]. The threshold for RAP was employed for the purpose of summarizing RAP as a measure over the entire recording period. Given RAP has a negative parabolic relationship with ICP, RAP values near 0 can mean both good and poor compensatory reserve, depending on the situation. As such, taking grand mean values of RAP over the entire recording period can be done, but their interpretation is extremely difficult, even during logistic regression analysis. This is why we have focused on the area under the RAP vs. time relationship, which provides a more objective and meaningful grand summary measure when talking about a summary metric over an entire recording period. This is not the case for wICP, as each minute the wICP value is calculated from the ICP and RAP value at that time, and is reflective of the physiologic situation.

### Statistics

All statistical analysis was conducted using R (R Core Team (2016). R: A language and environment for statistical computing. R Foundation for Statistical Computing, Vienna, Austria. URL https://www.R-project.org/) and XLSTAT (Addinsoft, New York, NY; https://www.xlstat.com/en/) add-on package to Microsoft Excel (Microsoft Office 15, Version 16.0.7369.1323). Normality of continuous variables was assessed via the Shapiro-Wilks test. For all testing described within, the alpha was set at 0.05 for significance.

Despite GOSE being collected at both 6 and 12 months post-injury in this cohort of patients, there was missing data present in both categories of outcome. Thus, we combined GOSE scores from both 6 and 12 months in order to provide a “6- to 12-month” GOSE. For patients where GOSE was reported for both 6 and 12 months, the superior GOSE score was selected for analysis. Both ICP and wICP were assessed across each ordinal category of GOSE using the Jonckheere-Terpstra test with 1000 permutations, assessing for statistically significant decreases in each ICP variable with increasing GOSE grade.

GOSE was then dichotomized into the following categories: (A) alive (GOSE 2 to 8) vs. dead (GOSE 1) and (B) favorable (GOSE 5 to 8) vs. unfavorable (GOSE 4 or less). Demographics and physiologic variables were compared between each dichotomized group via *t* test, Mann-Whitney *U*, and chi-square testing where appropriate. Box plots were created for variables of interest comparing between dichotomized groups.

Univariate logistic regression (ULR) was conducted, comparing variables to both dichotomized outcomes, assessing superiority using AUC and Delong’s test. Next, various multi-variable models were created, which included ICP, AMP, wICP, mean RAP, RAP AUC above 0 and RAP AUC above + 0.4. This study represented the first validation of the concept of wICP, and we therefore limited the complexity of the analyses undertaken. We only used univariate logistic regression, as we were primarily interested in validating previous single-center results in a multicenter dataset, to demonstrate the feasibility of such an approach. No adjustment for baseline characteristics was conducted, as study-wide imputation is currently being conducted to account for missing values.

## Results

### Patient demographics

There were 196 patients from the CENTER-TBI high-resolution ICU cohort, with high-frequency physiologic signals and complete demographic variables, which were included in this study. The mean age was 46.6 ± 19.7 years, with 150 being male. Median admission GCS was 8 (IQR 5 to 13), and mean duration of physiologic monitoring was 159.3 ± 115.1 h. All continuous variables were found to be non-parametrically distributed. Table 1 summarizes all of the patient demographics and base physiologic information.

**Table 1:** Patient demographics, physiology and outcome—entire population

		Mean/median (± sd or IQR)
Number of Patients		196
Age (years)		46.6 (19.7)
Sex	Male	150
	Female	46
Admission GCS (total)		8 (5 to 13)
Admission GCS (motor)		4 (2 to 6)
Admission pupil response	Bilaterally reactive	128
	Unilateral unreactive	17
	Bilaterally unreactive	51
Duration of high-frequency physiologic recording (hours)		159.3 (115.1)
ICP (mmHg)		14.3 (10.4)
AMP (mmHg)		2.6 (2.8)
CPP (mmHg)		69.6 (12.4)
wICP (mmHg)		5.8 (7.9)
RAP (a.u.)		0.614 (0.206)
RAP AUC—threshold of 0		5544.8 (4188.9)
RAP AUC—threshold of + 0.4		2625.8 (2158.6)
6- to 12-month GOSE		4 (2 to 6)
Number alive—6 to 12 months		149
Number dead—6 to 12 months		47
Number favorable outcome—6 to 12 months (GOSE 5 to 8)		94
Number unfavorable outcome—6 to 12 months (GOSE 1 to 4)		102

*AMP* pulse amplitude of ICP, *a.u.* arbitrary units, *AUC* integrated area under the RAP curve over time, *CPP* cerebral perfusion pressure, *GCS* Glasgow Coma Scale, *GOSE* Glasgow Outcome Score—Extended, *ICP* intracranial pressure, *IQR* inter-quartile range, *RAP* compensatory reserve index (moving correlation between AMP and ICP), *sd* standard deviation, *wICP* compensatory-reserve-weighted ICP (wICP = (1 − RAP) × ICP)

### Physiology and outcome

**Fig. 1:**
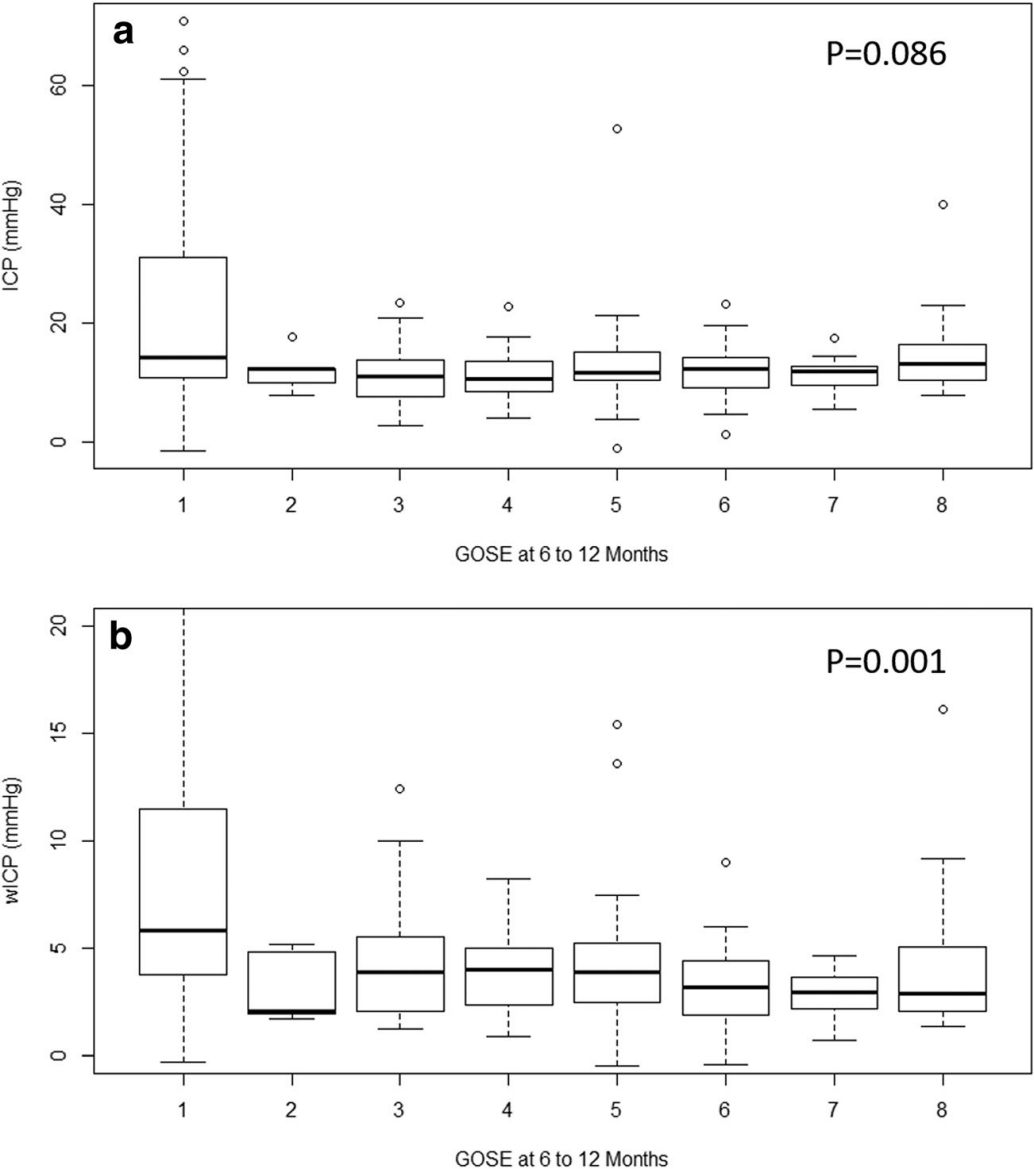
**a**, **b** ICP and wICP across GOSE categories. GOSE = Glasgow Outcome Score—Extended, ICP = intracranial pressure, mmHg = millimeters of Mercury, wICP = compensatory-reserve-weighted ICP (wICP = (1 − RAP) × ICP). *p values reported are for the Jonckheere-Terpstra test, which was set to assess for statistically significant decreases in mean values of ICP and wICP, with increase GOSE category

**Fig. 2:**
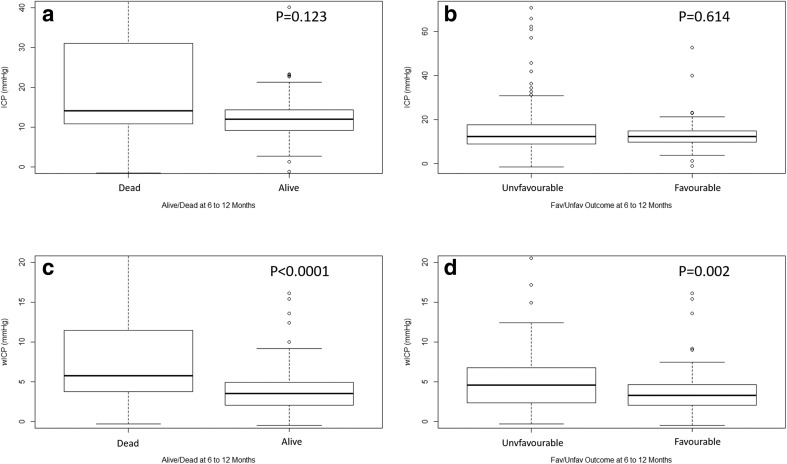
ICP and wICP across dichotomized 6 to 12 month outcomes. GOSE = Glasgow Outcome Score—Extended, ICP = intracranial pressure, mmHg = millimeters of mercury, wICP = compensatory-reserve-weighted ICP (wICP = (1 − RAP) × ICP). **a** Mean ICP for alive and dead outcomes. **b** Mean ICP for favorable and unfavorable outcomes. **c** Mean wICP for alive and dead outcomes. **d** Mean wICP for favorable and unfavorable outcomes. Alive/dead dichotomization (alive = GOSE ≥ 2, dead = GOSE 1). Favorable/unfavorable dichotomization (favorable = GOSE 5 to 8, unfavorable = GOSE 1 to 4). *p values reported are for Mann-Whitney *U* test, comparing mean values between dichotomized groupings

Appendix A summarizes the comparison of various patient demographics and physiology between the dichotomized 6- to 12-month outcome groups, using Mann-Whitney *U* and chi-square test where appropriate. Both mean age and mean wICP were noted to be significantly different between both alive/dead and favorable/unfavorable outcome groups with high mean age (*p* < 0.0001 for alive/dead, and *p* = 0.001 for favorable/unfavorable) and wICP (*p* < 0.0001 for alive/dead, and *p* = 0.002 for favorable/unfavorable) associated with worse outcomes. Mean ICP was not significantly different between either dichotomized group (*p* = 0.134 for alive/dead, and *p* = 0.614 for favorable/unfavorable). Figure 1 displays a box plot of ICP and wICP across each category of GOSE at 6 to 12 months, with wICP demonstrating a statistically significant decrease in mean value with increasing GOSE category via Jonckheere-Terpstra testing (*p* = 0.001). Mean ICP did not display a significant decrease with increasing GOSE score (*p* = 0.086). Figure 2 displays box plots of ICP and wICP across both dichotomized outcome groups, highlighting statistically significant lower mean wICP for both the alive and favorable outcome groups.

### Univariate logistic regression (ULR) analysis

Univariate logistic regression was performed for each demographic and physiologic variable with both 6- to 12-month dichotomized outcomes. Table 2 displays the results of the ULR analysis with AUCs and *p* values tabulated for each variable. From this analysis, it was found that age was statistically associated with both alive/dead (AUC = 0.737; 95% CI 0.652–0.737; *p* < 0.0001) and favorable/unfavorable (AUC = 0.680; 95% CI 0.605–0.754; *p* < 0.0001) outcomes, in keeping with prior literature [3, 10]. Mean ICP was only significantly associated with alive/dead (AUC = 0.642; 95% CI 0.538–0.746; *p* < 0.0001), not favorable/unfavorable outcome (AUC = 0.495; 95% CI 0.413–0.577; *p* = 0.059). wICP displayed a stronger association (i.e., larger AUC) with both alive/dead and favorable/unfavorable outcomes (AUC = 0.712; 95% CI 0.615–0.810; *p* = 0.0002, and AUC = 0.626; 95% CI 0.548–0.705; *p* = 0.015; respectively), compared to ICP. This stronger relationship between wICP and outcome held true even when comparing to models with ICP and RAP AUC.

**Table 2:** Univariate/bivariate logistic regression analysis for IMPACT core and physiologic variables

Variable	A/D AUC (95% CI)	*p* value	F/U AUC (95% CI)	*p* value
Age	0.737 (0.652–0.737)	*< 0.0001*	0.680 (0.605–0.754)	*< 0.0001*
Admission GCS Motor	0.559 (0.409–0.612)	0.160	0.576 (0.497–0.655)	*0.022*
Admission Pupil Reactivity	0.440 (0.336–0.524)	0.196	0.440 (0.372–0.507)	0.228
Mean ICP	0.642 (0.538–0.746)	*< 0.0001*	0.495 (0.413–0.577)	0.059
Mean AMP	0.671 (0.567–0.775)	*< 0.0001*	0.527 (0.445–0.609)	*0.045*
Mean RAP*	0.669 (0.575–0.762)	*0.002*	0.659 (0.582–0.735)	*0.0007*
Mean RAP AUC Above 0	0.619 (0.526–0.712)	*0.015*	0.495 (0.413–0.576)	0.359
Mean RAP AUC above + 0.4	0.635 (0.544–0.727)	*0.007*	0.539 (0.458–0.620)	0.137
wICP	0.712 (0.615–0.810)	*0.0002*	0.627 (0.548–0.705)	*0.015*
Multi-variable models				
ICP + RAP AUC Above 0	0.618 (0.525–0.711)	*< 0.0001*	0.495 (0.414–0.577)	0.126
ICP + RAP AUC above + 0.4	0.633 (0.541–0.724)	*< 0.0001*	0.538 (0.457–0.619)	0.069
ICP + AMP	0.657 (0.553–0.760)	*0.007*	0.508 (0.426–0.590)	0.222
ICP + AMP + mean RAP	0.653 (0.548–0.758)	*0.0001*	0.503 (0.421–0.586)	*0.001*

Italicized *p* values are those reaching significance (i.e., *p* < 0.05). *Despite these mean values of RAP demonstrate significant associations with the dichotomized outcomes, they are difficult to interpret given the nature of RAP. RAP values near 0 can mean both good and poor compensatory reserve, as such, during the averaging process to produce mean values over a recording period, it becomes difficult to interpret the meaning of such measures

*AMP* pulse amplitude of ICP, *A/D* alive/dead, *AUC* area under the receiver operating curve, *CI* confidence interval, *F/U* favorable/unfavorable outcome (i.e., favorable = Glasgow Outcome Scale of 5 to 8; unfavorable = Glasgow Outcome Scale of 1 to 4), *ICP* intra-cranial pressure, *IMPACT* International Mission for Prognosis and Analysis of Clinical Trials, *RAP* compensatory reserve index (moving correlation between AMP and ICP), *RAP AUC* integrated area under the RAP curve over time, *wICP* compensatory-reserve-weighted ICP (wICP = (1 − RAP) × ICP)

**Fig. 3:**
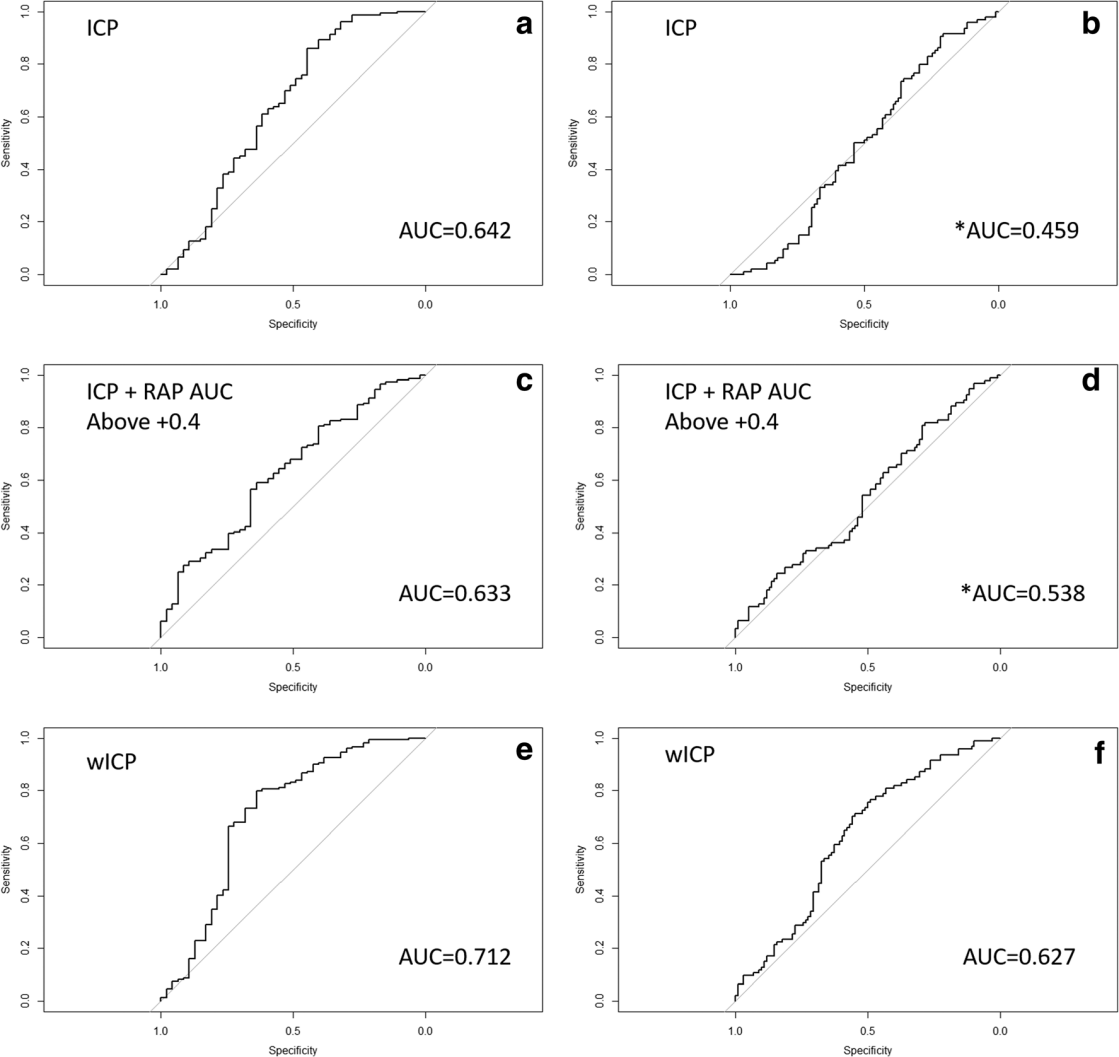
Univariate logistic regression—ICP, ICP + RAP AUC above + 0.4, and wICP receiver operating curves. GOSE = Glasgow Outcome Scale Extended, ICP = intracranial pressure, ULR = univariate logistic regression, wICP = compensatory-reserve-weighted ICP (wICP = (1 − RAP) × ICP). **a** ICP ULR for alive/dead outcome. **b** ICP ULR for favorable/unfavorable outcome. **c** ICP + RAP AUC above + 0.4 ULR for alive/dead outcome. **d** ICP + RAP AUC above + 0.4 for favorable/unfavorable outcome. **e** wICP ULR for alive/dead outcome. **f** wICP ULR for favorable/unfavorable outcome. Alive/dead dichotomization (alive = GOSE ≥ 2, dead = GOSE 1). Favorable/unfavorable dichotomization (favorable = GOSE 5 to 8, unfavorable = GOSE 1 to 4). *Indicates AUC reported failed to reach statistical significance in the ULR model

Comparing AUCs via Delong’s test indicated that there was no difference between the AUCs for alive/dead outcome association between ICP and wICP. However, wICP demonstrated a statistically significant higher AUC compared to ICP for favorable/unfavorable outcome (*p* = 0.002). Comparing the bivariate models with ICP and RAP AUC to the univariate model with wICP, for both alive/dead and favorable/unfavorable outcomes, the univariate models with wICP alone displayed statistically significant higher AUCs compared to the bivariate models with ICP and RAP AUC (*p* < 0.05 for all; Delong’s test). Figure 3 displays the receiver operating curves for ICP, ICP + RAP AUC above 0.4, and wICP.

Finally, comparing the univariate models with wICP to those containing (A) ICP + AMP and (B) ICP + AMP + mean RAP, the univariate models with wICP displayed statistically higher AUCs compared to both the ICP +AMP and the ICP +AMP + mean RAP models for favorable/unfavorable dichotomized outcomes (*p* < 0.05 for both; Delong’s test). However, there was no significant difference between models for the alive/dead dichotomization, despite a trend to higher AUC values for the univariate wICP model compared to both the ICP + AMP and ICP + AMP +RAP models. Table 2 summarizes all AUC, 95% confidence intervals, and *p* values for the multi-variable models.

## Discussion

Using data from a multicenter study, this manuscript confirms the conceptual basis for wICP measurement and demonstrates its ability to better discriminate mortality and functional outcome when compared to conventional ICP measurement in adult moderate/severe TBI. Two important aspects of our results deserve highlighting.

First, we have been able to confirm the association between wICP and mortality displayed in the previous publication on the topic using a multi-center data set. This provides validating evidence that wICP may provide important information, potentially beyond what ICP can provide, with lower wICP values associated with reduced mortality. This was exemplified in the stronger association between wICP and both dichotomized 6- to 12-month outcomes. While these data provide important insights into cerebrovascular physiology, the data do not, as yet, allow us to recommend adoption of wICP as a clinically used measure at this time.

Second, we have been able to display the strong association between wICP and favorable/unfavorable outcome, as demonstrated using ULR and during the multi-variable models tested. This is an important finding, as regardless of how wICP was compared to ICP, the statistical association between wICP and favorable/unfavorable outcome was in favor of wICP, with lower wICP values associated with favorable 6- to 12-month outcome. However, as with the association between wICP and mortality, these results should be considered preliminary and the use of wICP as a clinically monitored variable cannot be justified at this time.

### Limitations

Despite the promising results displayed, there are some important limitations to highlight. First, despite the data from the CENTER-TBI high-resolution cohort being collected in a prospective manner, the treatments and therapies received by the patients for their TBI remains heterogeneous, with both center-to-center and patient-by-patient variation. Such variation in treatment, complication profile, and hospital course could potentially impact the physiologic signals recorded. The potential impact of various ICU therapies on signal response should be emphasized, as this data does not represent the natural history of untreated cerebral physiology in moderate/severe TBI. Thus, ICP-directed treatments, amongst other therapies, may have impacted the physiology recorded. Despite that, the results portrayed in this study parallel those seen in the previous retrospective work on compensatory-reserve-weighted ICP. Furthermore, there exists the potential for within-patient variability of the physiologic signals over time, either in response to individual therapeutic measures, or based on individual physiologic differences between patients. This within-patient variability may have impacted the results seen within the preliminary results presented in this manuscript. As such, they require much further investigation and validation. Future analysis of wICP and RAP will need to account for this and may benefit from more complex time-series techniques, mixed effects, and latent class modelling.

Second, the overall patient numbers with outcome and basic demographics were low at 196. This high-resolution cohort was a small specialty sub-cohort within the larger CENTER-TBI data collection scheme. While extrapolation to other (or wider) populations of TBI patients remains unproven, we believe that the strength of statistically significant results displayed in this study is important, and warrant further exploration in larger multi-center TBI studies, where such high-frequency physiologic data is collected.

Third, we specifically did not adjust for baseline admission characteristics through multi-variable logistic regression analysis, given the CENTER-TBI study-wide imputation and validation processes for missing data is still ongoing and will be the focus of various other publications and studies, once completed. As such, we can only comment on univariate association with outcome at this time, as the focus was to provide a multi-center validation study for the previous retrospective single-center results from Cambridge. It is possible that through adjusting for baseline admission characteristics, the results may not be as significant. Such multi-variable models will be the focus of future studies from both CENTER-TBI and other high-resolution data sets.

Fourth, despite strong associations with global outcome at 6 to 12 months, and results indicating the wICP may be superior to ICP in outcome association, there are currently no treatment thresholds for wICP in TBI. This lack of treatment thresholds for wICP is in contrast to existing BTF suggested treatment thresholds for ICP. Such analysis of wICP thresholds will need to occur with larger, perhaps combined, prospective and retrospective high-frequency physiologic data sets in TBI patients.

Finally, wICP should still be considered an experimental variable despite the significant results in this study. To date, with this current study, only three works have evaluated compensatory-reserve-weighted ICP, all providing complementary results [2, 6]. Currently, wICP should not replace ICP in monitoring and care of moderate and severe TBI patients. Much further multi-center work is required to validate this measure as a clinically valuable physiologic parameter in TBI.

## Conclusions

Compensatory-reserve-weighted ICP displays superior outcome association over ICP for both alive/dead and favorable/unfavorable dichotomized outcomes at 6 to 12 months in adult TBI, through univariate analysis. Lower wICP is associated with better global outcomes. The results of this study provide multi-center validation of those seen in previous single-center studies. Further work is required to identify treatment thresholds for wICP in adult TBI.

## Electronic supplementary material

ESM 1 (DOCX 15 kb)
