# Supplementary material for: Compensatory-reserve-weighted intracranial pressure versus intracranial pressure for outcome association in adult traumatic brain injury: a CENTER-TBI validation study
Source: Acta Neurochir (Wien). 2019 May 3;161(7):1275–84. doi: 10.1007/s00701-019-03915-3 (PMC6581920; doi:10.1007/s00701-019-03915-3)
Supplement: Supplementary file 1 — (DOCX 15 kb) [file 701_2019_3915_MOESM1_ESM.docx]

*Appendix A: Summary of Patient Demographics Based on Alive/Dead or Favourable/Unfavourable Outcome Groups – Mann U and Chi-Square Testing*

| **Variable** | **Mean/Median Value (+/-sd or IQR)** | | **p-value** | **Mean/Median Value (+/-sd or IQR)** | | **p-value** |
| --- | --- | --- | --- | --- | --- | --- |
|  | **Alive** | **Dead** |  | **Favourable** | **Unfavourable** |  |
| **Number of Patients** | 149 | 47 |  | 94 | 102 |  |
| **Age** | 43.4 (18.9) | 57.0 (19.2) | **<0.0001** | 40.9 (17.6) | 51.4 (20.4) | **0.001** |
| **Sex (# Male)** | 27 | 104 | 0.090 | 70 | 61 | 0.696 |
| **Admission GCS (Total)** | 8 (5 to 13) | 8 (3 to 13) | 0.778 | 8 (6 to 13) | 6 (4 to 12) | 0.062 |
| **Admission GCS Motor** | 4 (1 to 6) | 4 (2 to 5) | 0.992 | 4 (1 to 6) | 5 (3 to 6) | 0.035 |
| **Admission Pupil Status** | * | * | >0.05 | * | * | >0.05 |
| **Length of Recording (hours)** | 165.3 (120.5) | 140.1 (94.5) | 0.070 | 153.6 (121.1) | 164.5 (110.0) | 0.378 |
| **Mean ICP (mm Hg)** | 12.3 (6.0) | 20.6 (16.9) | 0.134 | 13.0 (6.6) | 15.6 (12.8) | 0.614 |
| **Mean AMP (mm Hg)** | 2.1 (1.2) | 4.4 (4.9) | **0.001** | 2.1 (1.3) | 3.0 (3.6) | 0.521 |
| **Mean CPP (mm Hg)** | 71.4 (9.3) | 63.9 (18.2) | 0.175 | 71.5 (9.6) | 67.9 (14.4) | 0.650 |
| **Mean wICP (mm Hg)** | 4.1 (3.8) | 11.1 (13.4) | **<0.0001** | 4.1 (4.4) | 7.3 (9.8) | **0.002** |
| **Mean RAP (a.u.)** | 0.641 (0.197) | 0.528 (0.212) | **0.001** | 0.668 (0.194) | 0.575 (0.205) | 0.885 |
| **Mean RAP AUC Above 0** | 5955.5 (444.4) | 4143.0 (2953.9) | **0.014** | 5831.7 (4795.4) | 5280.4 (3543.4) | 0.348 |
| **Mean RAP AUC Above +0.4** | 2860.0 (2286.5) | 1883.3 (1480.2) | **0.005** | 2868.1 (2484.3) | 2402.5 (1791.2) | **0.002** |

*AMP = pulse amplitude of ICP, a.u. = arbitrary units, AUC = integrated area under the RAP over time curve, CPP = cerebral perfusion pressure, GCS = Glasgow Coma Scale, ICP = intra-cranial pressure, IQR = inter-quartile range, mm Hg = millimetres of Mercury, RAP = compensatory reserve index (moving correlation between AMP and ICP), sd = standard deviation, wICP = compensatory reserve weighted ICP (wICP = (1-RAP)*ICP), RAP = compensatory reserve index (moving correlation between AMP and ICP), wICP = compensatory reserve weighed ICP (wICP = (1-RAP)*ICP).. Note: all bolded p-values are those <0.05 when comparing the variables between Alive/Dead and Favourable/Unfavourable outcome groups. Favourable = Glasgow Outcome Scale of 5 to 8, Unfavourable = Glasgow Outcome Scale of 1 to 4. *No statistically significant difference for number of patients with bilaterally reactive, unilateral reactive, or bilaterally unreactive pupils between both Alive/Dead and Favourable/Unfavourable groups.*
